# Retraction: MiR200b regulates autophagy associated with chemoresistance in human lung adenocarcinoma

**DOI:** 10.18632/oncotarget.28886

**Published:** 2026-05-20

**Authors:** Banzhou Pan, Bing Feng, Yitian Chen, Guichun Huang, Rui Wang, Longbang Chen, Haizhu Song

**Affiliations:** ^1^Department of Medical Oncology, Jinling Hospital, School of Medicine, Nanjing University, Nanjing 210002, China; ^*^These authors have contributed equally to this work

**This article has been retracted:** Oncotarget has concluded its investigation of this paper, and the following issues were identified:

- Figure 1C: Identical cells were found in panels representing different treatments. Specifically, SPC-A1/DTX cells transfected with PmiR-200b and treated with Docitoxel (DTX) or Cisplatin (Cis) share a cell image. Additionally, the PmiR-NC/Cis image is duplicated in Figure 2G (H1299/DTX cells transfected with pDsRed1-Control). Furthermore, the image of SPC-A1 cells transfected with AmiR-NC and treated with DTX contains a cell identical to the Cisplatin treatment image.
- Figure 4B: In the SPC-A1/DTX colony formation assay, plates 3 and 4 in the control row are duplicates of plates 1 and 4 in Figure 4D of a previous publication by the same group [1], despite representing a different cell type (H1299/DTX).
- Figure 5A: Flow cytometry plots 1 and 4 in the Cisplatin row are duplicates.
- Figure 6C: Ki67 immunohistochemistry images for pcDNA miR-NC tumors are duplicates of the PCNA IHC images for two control shRNA tumors in Figure 8C of the aforementioned article [1].

The authors did not respond to requests for comment or provide the original data. Consequently, the editorial decision has been made to retract this paper. Oncotarget has reached out to all authors to notify them of this retraction but has received no response.

Original article: Oncotarget. 2015; 6:32805–32820. 32805-32820. https://doi.org/10.18632/oncotarget.5352
